# The Coexistence of Common Pulmonary Diseases on the Histologic Type of Lung Cancer in Both Genders in Taiwan: A STROBE-Compliant Article

**DOI:** 10.1097/MD.0000000000000127

**Published:** 2014-12-12

**Authors:** Zhi-Hong Jian, Chia-Chi Lung, Jing-Yang Huang, Pei-Chieh Ko, Shiou-Rung Jan, Oswald Ndi Nfor, Wen-Yuan Ku, Chien-Chang Ho, Hui-Hsien Pan, Yung-Po Liaw

**Affiliations:** From the Department of Public Health and Institute of Public Health (ZHJ, CCL, JYH, PCK, SRJ, ONN, WYK, YPL); Department of Family and Community Medicine, Chung Shan Medical University Hospital, Taichung City (CCL, YPL); Department of Physical Education (CCH), Fu Jen Catholic University, New Taipei City, Taiwan; Department of Pediatrics (HHP); and School of Medicine, Chung Shan Medical University Hospital, Taichung City, Taiwan (HHP).

## Abstract

Effects of pulmonary diseases [asthma, chronic obstructive pulmonary disease (COPD), and lung tuberculosis (TB)] on subsequent lung cancer development have been reported. However, whether patients with coexisting pulmonary diseases are at greater risk of developing various histologic types of lung cancer remains elusive.

Patients newly diagnosed with lung cancer between 2004 and 2008 were identified from National Health Insurance Research Database (Taiwan). The histologic types of lung cancer were further confirmed using Taiwan Cancer Registry Database. Cox proportional hazard regression was used to calculate the hazard ratio (HR) of coexisting asthma, COPD and/or TB to estimate lung cancer risk by histologic type.

During the study period, 32,759 cases of lung cancer were identified from 15,219,024 residents age 20 years and older, who were free from the disease before 2003. Coexisting pulmonary diseases showed stronger association with lung cancer than specific lung disorders. Specifically, among men, the HRs for squamous cell carcinoma (SqCC) were 3.98 (95% CI, 3.22–4.93), 2.68 (95% CI, 2.45–2.93), and 2.57 (95% CI, 2.10–3.13) for individuals with asthma+COPD+TB, asthma+COPD, and COPD+TB, respectively. Among women, the HRs for SqCC were 3.64 (95% CI, 1.88–7.05), 3.35 (95% CI, 1.59–7.07), and 2.21 (95% CI, 1.66–2.94) for individuals with TB, COPD+TB, and asthma+COPD, respectively. Adenocarcinoma HRs for men and women were 2.00 (95% CI, 1.54–2.60) and 2.82 (95% CI, 1.97–4.04) for individuals with asthma+COPD+TB, 2.28 (95% CI, 1.91–2.73) and 2.16 (95% CI, 1.57–2.95) for COPD+TB, and 1.76 (95% CI, 1.04–2.97) and 2.04 (95% CI, 1.02–4.09) for individuals with asthma+TB. Specifically, small cell carcinoma (SmCC) HRs among men were 3.65 (95% CI, 1.97–6.80), 2.20 (95% CI, 1.45–3.36), and 2.14 (95% CI, 1.86–2.47) for those with asthma+TB, asthma+COPD+TB, and asthma+ COPD, respectively. Among women, the HRs of SmCC were 8.97 (95% CI, 3.31–24.28), 3.94 (95% CI, 1.25–12.35) and 3.33 (95% CI, 2.23–4.97) for those with asthma+COPD+TB, COPD+TB, and asthma+COPD, respectively.

Patients with coexistence of pulmonary diseases were more susceptible to lung cancer. Affected persons deserve greater attention while undergoing cancer screening.

## INTRODUCTION

Lung cancer is the second leading disease contributing to years of life lost in the United States.^1^ Recent evidence suggested that chronic inflammation may be involved in lung carcinogenesis.^2^ Among lung diseases with chronic inflammation, asthma,^3^ chronic obstructive pulmonary disease (COPD),^4,5^ and pulmonary tuberculosis (TB)^6^ have been associated with lung cancers. Asthma increases the risks of squamous cell carcinoma (SqCC) and small cell carcinoma (SmCC).^7^ COPD is a risk factor of the SqCC histological subtype.^8^ TB has been independently associated with an elevated risk of SqCC, SmCC, and adenocarcinoma.^9^ A direct association between TB and adenocarcinoma was found in nonwesternized countries.^10^ Lung cancer risks increased greatly in patients with coexisting TB and COPD.^11^

To characterize the relationship between coexisting pulmonary diseases (COPD, asthma, and/or TB) and risk of developing various histologic types of lung cancer, a population-based cohort study is highly desirable but has rarely been conducted. In this study, we conducted a cohort study in Taiwanese population using the National Health Insurance Research Database (NHIRD) with a follow-up period of 5 years.

## METHODS

### Data Source

This study used generalized data retrieved from NHIRD between 2004 and 2008. Taiwan's National Health Insurance covers >99% of the 23 million residents and consists of enrollment files, claims data, catastrophic illness files, and registry for treatments. The NHIRD is one of the largest datasets and has been described in detail in previous studies.^12,13^ The data were used to measure patients’ demographics and comorbidities. This study was designed to assess whether individuals with coexisting pulmonary diseases were associated with an increased risk of developing various histologic types of lung cancer, using combined datasets: NHIRD, Taiwan Cancer Registry Database (TCRD), and National Death Registry Database. All datasets were encrypted and were strictly confidential. This study has been approved by the institutional review board of the Chung-Shan Medical University Hospital, Taiwan.

### Identification of Patients with Lung Cancer

Individuals age 20 years and older who were free of lung cancer before 2003 were enrolled from NHIRD. Individuals with incomplete information, such as sex and registry data were excluded. The study began in 2004 to count cases with newly diagnosed lung cancer and follow until death, loss of follow-up, or the end of 2008. The lung cancer was identified using the International Classification of Diseases, Ninth Revision, Clinical Modification (ICD-9-CM) code 162 for lung cancer.

The histologic types of lung cancer were further confirmed by TCRD. All major cancer care hospitals in Taiwan are obligated to submit cancer type, initial tumor stages and histology to the Taiwan Cancer Registry established by the Bureau of Health Promotion, Department of Health since 1979 to monitor the incidence and the mortality rates of cancer.^14^ The histological types of lung cancer were biopsy proven. Lung cancer was coded by ICD-9-CM 162 or ICD 10 C34.0, C34.1, C34.2, C34.3, C34.8, and C34.9 in TCRD. Morphological diagnoses were coded by using the ninth revision of the International Classification of Diseases for Oncology (ICD-O); based on ICD-O codes 80522, 80523, 80702, 80703, 80713, 80723, 80733, 80743, 80763, 80823, 80833, and 80843 for lung SqCC, codes 80503, 81402, 81403, 81413, 81433, 82113, 82503, 82513, 82523, 82553, 82603, 83103, 83233, 84603, 84803, 84813, 84903, and 85003 for adenocarcinoma, code 80023, 80412, 80413, 80423, 80433, 80453, and 94733 for SmCC, and code 80123, 80143, 80203, 80213, 80303, and 80313 for large cell carcinoma.

The National Death Registry Database, causes of death classified by ICD-9-CM, was then linked to the NHIRD and TCRD to assess the age of onset accurately, estimate person-year follow-up, confirm death and survival time, and minimize potentially unconﬁrmed cancer diagnoses in this study cohort.

### Statistical Analysis

All statistical analyses were performed using the SAS statistical package (Version 9.3; SAS Institute, Inc., Cary, NC). Descriptive statistical analyses were conducted using chi-square test to compare the differences in sociodemographic characteristics and comorbidities between the patients with lung cancer and controls. To evaluate the age and gender effects, we classified study individuals by age according to the following categories: 20 to 39, 40 to 59, 60 to 79, and ≥80 years and by gender. Potential confounding factors were listed according to established risk factors, and analyses were performed to establish whether these variables were substantially associated with histologic type of lung cancer. The diagnoses of pulmonary diseases and comorbidities were confirmed if individuals had 2 or more outpatient visits or one-time admission in 1 year between 2001 and 2003. Baseline pulmonary diseases and comorbidities were diagnosed as follows: asthma (ICD-9-CM: 493), COPD (ICD-9-CM: 490, 491, 492, 494, 496), and TB (ICD-9-CM: 010, 011, 012, 137.0). They are characterized by chronic airway inflammatory processes and have been associated with lung cancers.^3,5,6^ Patients with chronic renal disease have increased risk of lung cancer (ICD-9-CM: 585, 586).^15^ The relationship between diabetes mellitus (ICD-9-CM: 250, excluding type 1 DM) and lung cancers has been reported.^16^ Hyperlipidemia (ICD-9-CM: 272) as part of the metabolic syndrome is thought to be linked to cancer risk.^17^ Lifestyle behavior such as smoking, was not recorded in the NHIRD, hence, preventing direct adjustment of possible confounders. Smoking increases the chances of developing COPD and smoking-related cancers. Therefore, to investigate the potential impact of smoking, we also adjusted for smoking-related cancers such as lip, oral cavity, nasal cavity, pharynx, larynx, and esophagus (ICD-9-CM: 140–150, 160–161), pancreas (ICD-9-CM: 157), kidney, and urinary cancer (ICD-9-CM: 189).^11^ To determine the strength of coexisting pulmonary diseases and various histologic types of lung cancer, multivariable analyses and stratified analyses using hazard ratio (HR) were carried out with Cox proportional hazards models. Statistical signiﬁcance level was reached at *P* < 0.05.

## RESULTS

A total of 17,859,318 Taiwan residents age 20 years and older were enrolled in this study. We excluded individuals diagnosed with lung cancer before 2003 (n = 39,623) as well as persons with incomplete data on sex (n = 2,600,565), registration (n = 5), and presence of death (n = 101). Finally, 15,219,024 cohorts (8,002,536 males and 7,216,488 females) were followed up from 2004 to 2008. During the study period, 32,759 cases of lung cancer were identified. The diagnoses of histologic types were as follows: adenocarcinoma, 47.3% (male/female cases: 8778/6712), SqCC, 20.3% (male/female: 5877/760), SmCC, 9.2% (male/female: 2751/268), large cell carcinoma, 0.7% (male/female: 183/57), and others, 23.2% (male/female cases: 5283/2090). Demographic characteristics and comorbidities of the study population are summarized in Table 1. Compared with controls, the highest rates of pulmonary diseases, hyperlipidemia, diabetes, chronic renal disease, and smoking-related cancers were observed in patients with lung cancer.

**TABLE 1:**
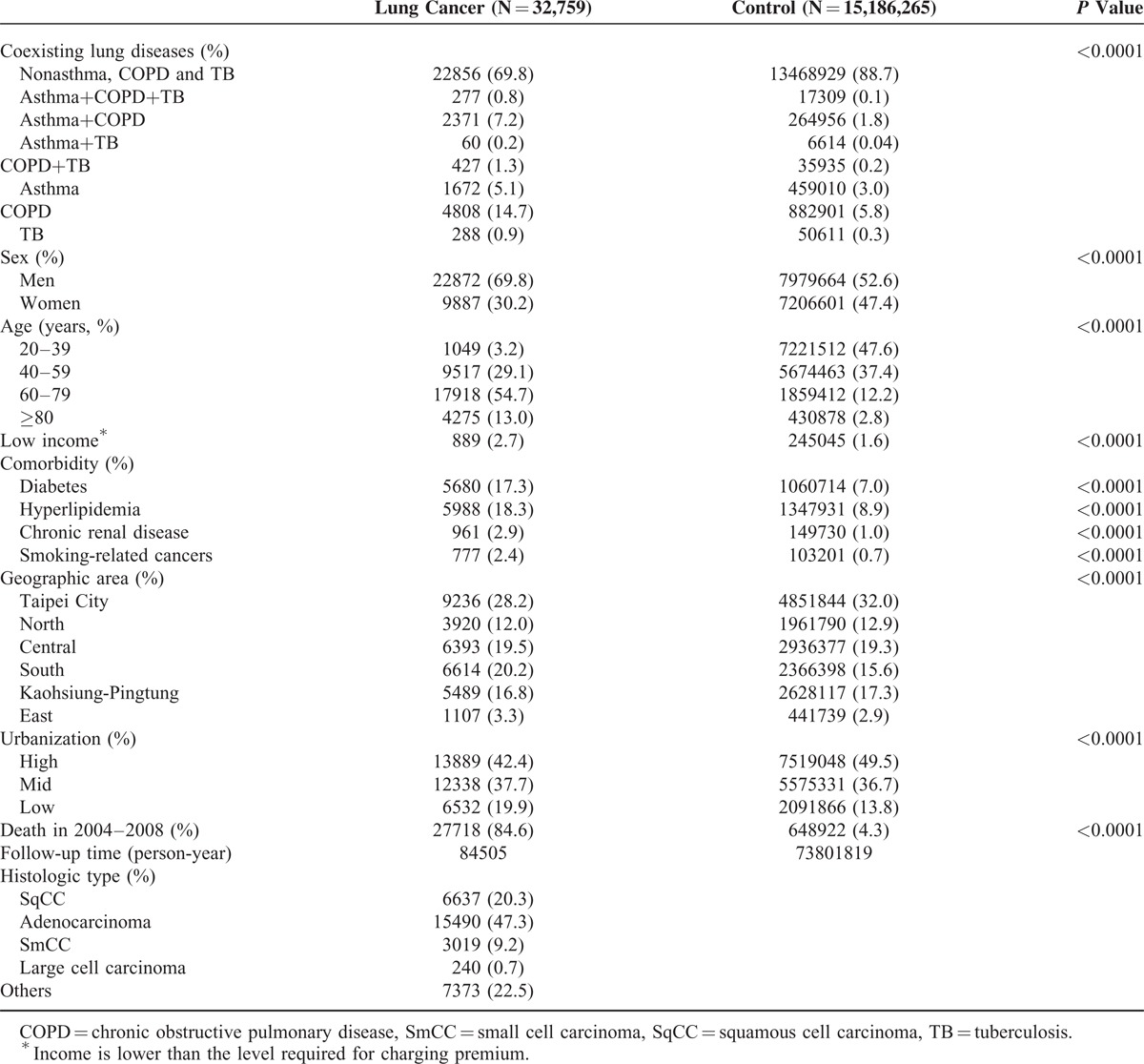
Characteristics of the Study Population

In Table 2, Cox regression analysis showed that the risk of lung cancer was significantly higher in male patients with asthma+COPD+TB (HR, 2.81; 95% confidential interval [CI], 2.46–3.20], COPD+TB (HR, 2.42; 95% CI, 2.18–2.69), asthma+COPD (HR, 2.21; 95% CI, 2.11–2.32), and asthma+ TB (HR, 2.12; CI, 1.59–2.83) after adjusting for low income, age, comorbidities, urbanization, and geographical area. There was an increased risk of lung cancer among females with asthma+COPD+TB, COPD+TB, asthma+TB, and asthma+COPD whose HRs and CIs were 2.96 (95% CI, 2.24–3.93), 2.41 (95% CI, 1.90–3.07), 2.21 (95% CI, 1.28–3.81), and 1.64 (95% CI, 1.50–1.79), respectively.

**TABLE 2:**
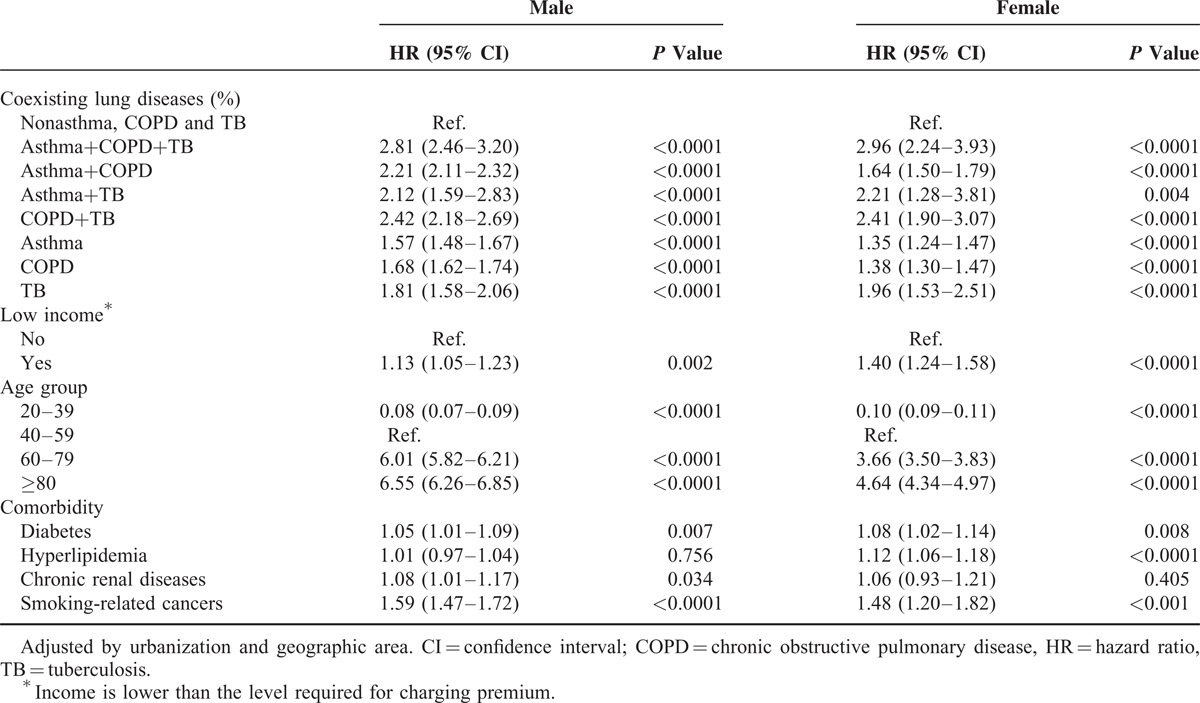
HRs and 95% CIs of Lung Cancer by Sex

Table 3 illustrates the coexisting pulmonary diseases and SqCC risk according to gender. Compared with those without pulmonary disease, the HRs of SqCC were higher in men with asthma+COPD+TB (HR, 3.98; 95% CI, 3.22–4.93), asthma+COPD (HR, 2.68; 95% CI, 2.45–2.93), COPD+TB (HR, 2.57; 95% CI, 2.10–3.13), and asthma+TB (HR, 2.01; 95% CI, 1.14–3.54). The HR for SqCC in women with TB, COPD+TB, and asthma+COPD were 3.64 (95% CI, 1.88–7.05), 3.35 (95% CI, 1.59–7.07), and 2.21 (95% CI, 1.66–2.94), respectively. The same association was not found among females with asthma+TB due to the small sample size.

**TABLE 3:**
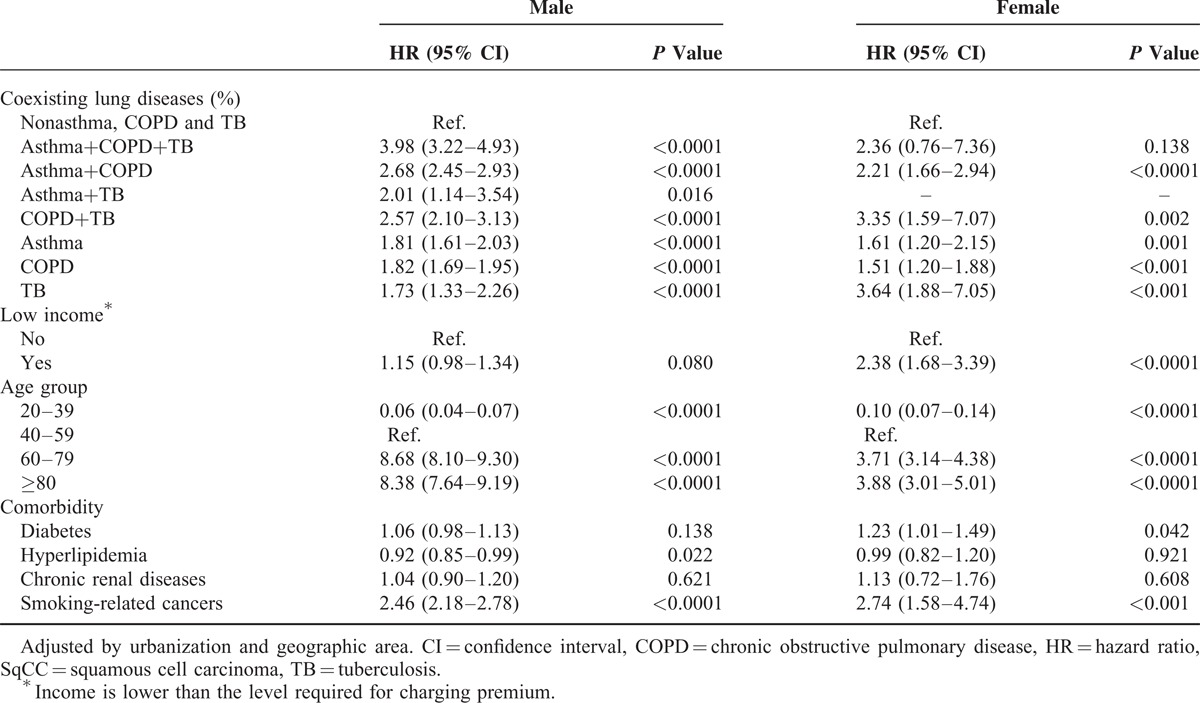
HRs and 95% CIs of SqCC by Sex

Table 4 illustrates the HR of lung adenocarcinoma associated with coexisting pulmonary diseases according to gender. The HRs of adenocarcinoma were higher in men with COPD+TB (HR, 2.28; 95% CI, 1.91–2.73), asthma+COPD+TB (HR, 2.00; 95% CI, 1.54–2.60), asthma+TB (HR, 1.76; 95% CI, 1.04–2.97), and asthma+COPD (HR, 1.71; 95% CI, 1.57–1.88). The HR for adenocarcinoma in women with asthma+COPD+TB, COPD+TB, asthma+TB, and asthma+COPD were 2.82 (95% CI, 1.97–4.04), 2.16 (95% CI, 1.57–2.95), 2.04 (95% CI, 1.02–4.09), and 1.53 (95% CI, 1.37–1.72), respectively.

**TABLE 4:**
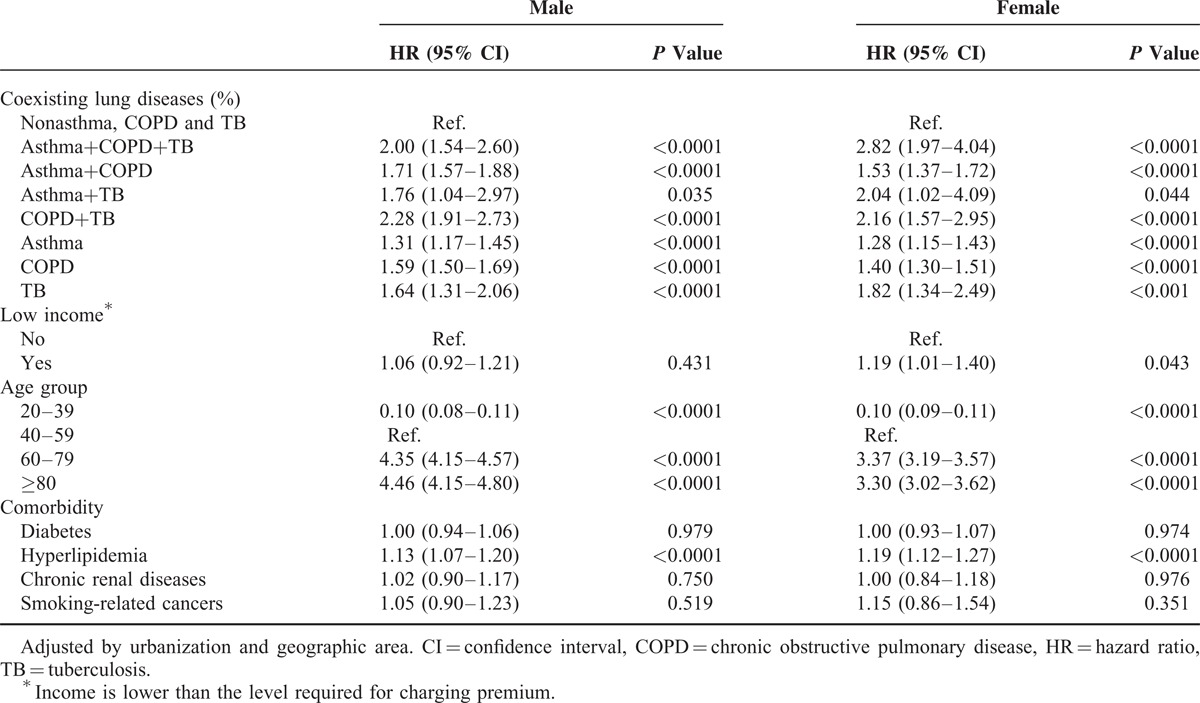
HRs and 95% CIs of Adenocarcinoma by Sex

Table 5 shows coexisting pulmonary diseases and SmCC risk by gender. The HRs for SmCC were higher in men with asthma+TB (HR, 3.65; 95% CI, 1.97–6.80), asthma+COPD+TB (HR, 2.20; 95% CI, 1.45–3.36), asthma+COPD (HR, 2.14; 95% CI, 1.86–2.47), and COPD+TB (HR, 2.08; 95% CI, 1.50–2.87). Women with asthma+COPD+TB, COPD+TB, and asthma+COPD had the highest risk of SmCC. Their HRs and CIs were 8.97 (95% CI, 3.31–24.28), 3.94 (95% CI, 1.25–12.35), and 3.33 (95% CI, 2.23–4.97), respectively.

**TABLE 5:**
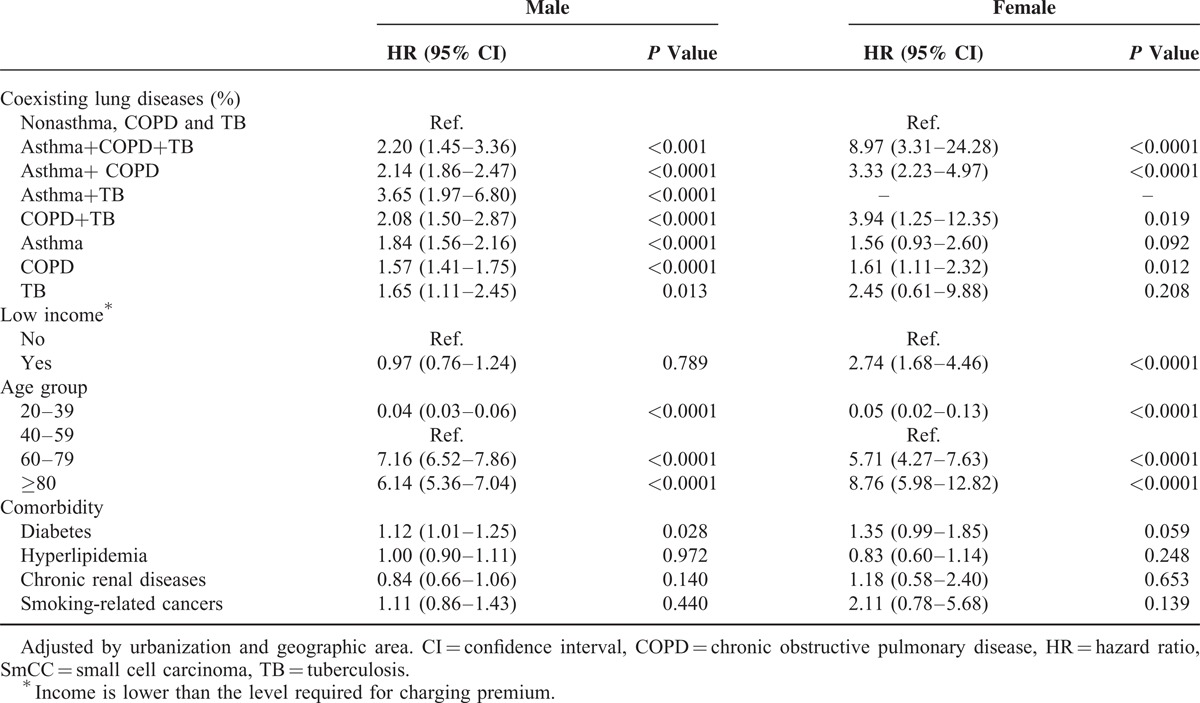
HRs and 95% CIs of SmCC by Sex

## DISCUSSION

Over the past decade, some studies have documented a possible link between lung cancer and common pulmonary diseases, including asthma, COPD, and TB.^3,6,8^ However, the influence of coexisting pulmonary diseases on histologic types of lung cancer has not been addressed. The most important finding in this study is that, unlike specific lung disorders, coexisting pulmonary diseases showed stronger association with lung cancer.

The prevalence of smoking in Taiwan is 45.7% in men and 4.8% in women.^18^ Obviously, the effect of smoking on lung cancer could not be fully explained. The odds ratio of lung cancer ranged from 1.28 to 4.78 in nonsmoking patients with asthma.^7,19–21^ The strengths of the associations found in this study are consistent with those reported previously. However, very few studies have documented the association in different histologic types of lung cancer. In a population-based cohort study conducted in Sweden, the risk of lung cancer in patients with asthma was higher for SqCC and SmCC than for adenocarcinoma.^22^ A meta-analysis showed that asthma increased risks of SqCC, relative risk (RR): 1.69 (95% CI, 1.26–2.26) and SmCC, RR of 1.71 (95% CI, 0.99–2.95) unlike adenocarcinoma, which showed a weaker association.^7^ In this study, asthma was associated with increasing risk of the 3 histologic types of lung cancer in both genders.

COPD has been associated with the occurrence of lung cancer.^23^ Cigarette smoking is so strongly associated with both COPD and lung cancer that it is hard to remove the effect of smoking by presuming statistical adjustment procedures.^24^ A previous history of COPD conferred a RR of 2.22 (95% CI, 1.66–2.97), but the effect was attenuated in nonsmokers.^25^ There was an important question whether the relationship between lung cancer and COPD was subtype specific. COPD is a risk factor for the SqCC among smokers.^8^ In this study, COPD is associated with 3 histological types of lung cancer.

A meta-analysis showed that TB conferred a RR of 1.90 (95% CI, 1.45–2.50) for lung cancer when restricting analysis to nonsmokers.^25^ Among both genders, nonsmokers with TB had significant association with SqCC and adenocarcinoma, whereas male and female smokers with TB were associated with SqCC, SmCC, and adenocarcinoma (men) and adenocarcinoma (women).^26^ Yu et al^11^ conducted a cohort of 1 million residents and showed that there was an increased risk of lung cancer in TB with an adjusted HR of 3.32 (95% CI, 2.70–4.09), stronger than COPD (HR, 2.30; 95% CI, 2.07–2.55). In a hospital-based case–control study, nonsmoking female patients with a history of TB (adjusted odds ratio = 4.7; 95% CI, 1.6–13.2) experienced greater risk of lung cancer, specifically adenocarcinoma and SqCC, than asthma and COPD.^27^ In a meta-analysis, COPD conferred a RR of 2.22 (95% CI, 1.66–2.97) and its effects were attenuated when restricting analysis to nonsmokers only for COPD (RR = 1.22; 95% CI, 0.97–1.53), however, remained significant for TB 1.90 (95% CI, 1.45–2.50).^25^ In this study, the HR for TB was higher than for asthma+COPD, asthma and COPD in all cases of lung cancer and SqCC among women. Thus, TB appeared to have a greater effect on lung cancer among women compared with asthma or COPD. Further studies are necessary to elucidate such mechanisms.

Asthma is a common chronic inﬂammatory airway disease that affects 300 million people of all ages and all ethnic backgrounds worldwide.^28^ The prevalence of asthma in Taiwan is 11.9 %.^29^ The average annual prevalence and incidence rates of COPD have been reported 2.48/100 and 0.66/100.^30^ TB remains a major public health problem in Taiwan and a total of 57,405 new TB cases were diagnosed from 2005 to 2007.^31^ It is particularly important in Taiwan where the prevalence of asthma, COPD, and TB is high. Wang et al conducted a case–control study in which nonsmoking women who had more than 1 previous lung disease tended to be at higher risk of lung cancer than those with only 1 of them.^20^ Although the additive combined effect of pulmonary diseases varied according to histologic type, coexistence of 2 or more pulmonary diseases had a significantly increased risk for the 3 histologic types of lung cancer. Biologically, the additive effects between coexisting pulmonary diseases and histologic types may be explained by compromised immune clearance of *Mycobacterium tuberculosis* and chronic inflammatory processes of the lung that predisposes to malignant transformation.^32,33^

Comorbidities have been reported to be associated with lung cancer mortality.^34^ Coexisting COPD is associated with worse survival outcomes of lung cancer for men and for SqCC type. There was also an increased risk of lung cancer mortality in patients with asthma.^35^ Asthma and COPD may coexist in the same patients and asthma–COPD overlap syndrome is an important clinical phenotype. The prevalence of asthma–COPD overlap syndrome in Italy was 1.6%, 2.1%, and 4.5% in the 20 to 44, 45 to 64, and 65 to 84 age groups.^36^ Patients with overlap syndrome have worse lung function, a worse quality of life, more severity and frequency of respiratory exacerbations, and increased mortality and health care utilization than those with asthma or COPD alone. ^37–39^ However, the survival in patients with coexisting TB and lung cancer remains controversial.^40,41^

The strengths of this study were numerous. First, our study was a prospective cohort study that included large sample size and long follow-up. In addition, the temporal relationship between coexisting pulmonary diseases and histologic types is difficult to evaluate in case–control studies. Small sample size limited reliability of previous study and did not permit gender-specific analysis of risk factors separately by histology. Second, there was completeness of cancer case ascertainment, hence, allowing little possibility for recall and selection bias. Nevertheless, this study had some limitations. First, detection bias might have been possible because of frequent hospital visits, hence, leading to a higher detection rate of early-stage lung cancer. Second, asthma, COPD, and TB patients may have taken more medications that may have complicated the situation. This study did not evaluate the effects of medications. Third, NHIRD does not contain detailed information regarding smoking history, radon exposure, occupational exposures, diet preference, and family history,^20^ all of which may be risk factors for lung cancer. Smoking is an important confounding factor of lung cancer and we used COPD and smoking-related cancers to substitute smoking as one of covariates in the adjustment measures.

In conclusion, we found that coexisting pulmonary diseases conferred a higher risk of lung cancer than exposure to one of the diseases. Because of the aging population and the increase in prevalence of asthma, COPD, and TB, cancer screening is recommended for patients with coexisting pulmonary diseases.
